# Sensory Perceptions, Evoked Emotions, and Instrumental Parameters of Plant‐Based and Hybrid Meatballs Added With Fermented Cashew Apple Waste: A Multifactorial Approach

**DOI:** 10.1111/1750-3841.71462

**Published:** 2026-09-21

**Authors:** Natália Ferreira Negreiros, Rogério Silva de Almeida, Tatiana Colombo Pimentel, Mercia de Sousa Galvão, Tânia Beatriz Santos Cândido, Leila Moreira de Carvalho, Normando Mendes Ribeiro‐Filho, Lary Souza Olegario, Taliana Kênia Alencar Bezerra, Marta Suely Madruga, Fábio Anderson Pereira da Silva

**Affiliations:** ^1^ Post‐Graduate Program in Food Science and Technology, Department of Food Engineering, Technology Center Federal University of Paraíba João Pessoa Brazil; ^2^ Federal Institute of Paraná Paranavaí Brazil; ^3^ Department of Food Engineering, Technology Center Federal University of Paraíba João Pessoa Brazil; ^4^ Department of Management and Agro‐Industrial Technology, Center for Humanities, Social and Agrarian Sciences Federal University of Paraiba Bananeiras Brazil; ^5^ IPROCAR Research Institute University of Extremadura Cáceres Spain

**Keywords:** alternative proteins, analogs, fermentation, instrumental profile, sensory techniques

## Abstract

Growing demand for proteins has driven exploration of unconventional plant matrices combined with fermentation technologies. Cashew apple bagasse, a byproduct of juice production, is a potential fermentation substrate for microbial protein production (single‐cell protein). This study evaluated the effects of 10%, 20%, and 30% cashew bagasse flour enriched with *Saccharomyces cerevisiae* protein on the instrumental properties (proximate composition, color, texture, and volatile compound profile), sensory profile (Preferred Attribute Elicitation [PAE] and Free Choice Profiling [FCP]), and consumers’ emotional responses (using a validated food‐specific emoji list) to plant‐based and hybrid meatballs. Incorporation at 30% significantly increased hardness and gumminess while reducing elasticity in all samples (*p* < 0.05), with more pronounced changes in hybrid meatballs. The enriched flour increased volatile complexity, introducing ester compounds in hybrid products and imparting a “fermented” character in analogs, which masked and/or reduced terpene contribution. The L* value increased significantly in hybrid products, from 26.08 in the control to 30.85 at 30% incorporation, indicating greater color lightness (*p* < 0.05). In descriptive tests, consumers perceived intensity differences among products but described them using similar terms, generating 19 attributes in the check‐all‐that‐apply (CATA) test. Acceptance thresholds were 20% incorporation for plant‐based analogs and 10% for hybrid products, influenced by familiarity with meat matrices and lack of reference standards for plant‐based foods. Regarding emotional profiling, enriched flour had a greater impact than product type, reducing positive emotions and increasing feelings of confusion and surprise, without leading to rejection. Consumer responses varied by the base matrix (plant or animal), while moderate incorporation levels maintained technological and sensory balance.

## 1 Introduction

Human diets have evolved throughout history under the influence of cultural, social, and environmental factors, alongside growing concerns about health and sustainability (Biasini et al. 2021). The association between high consumption of animal‐derived foods and negative health and environmental outcomes has driven interest in diets with greater plant‐based content (Zandstra et al. 2024). In response, the food industry has intensified the development of meat alternatives, including plant‐based meat analogs produced entirely from plant matrices and hybrid products that partially replace meat with plant ingredients. These approaches reduce meat consumption in line with sustainability goals while potentially improving nutritional profiles (Olegario et al. 2025; Ishaq et al. 2022).

Legumes are the primary plant protein sources used in meat analogs and hybrid products due to their high protein content, with soy being the most widely used raw material (Messina et al. 2023). However, soy presents notable limitations, including high allergenic potential and environmental impacts (Pi et al. 2021; Salvagiotti et al. 2024; Ren et al. 2025). This has directed research toward alternative proteins and circular economy strategies, particularly the valorization of agro‐industrial residues and byproducts (Zié et al. 2025). Cashew apple bagasse stands out in this context as a source of nutritional and bioactive compounds that can be valorized through extraction, fermentation, and incorporation into food formulations, contributing to their nutritional, functional, and sensory properties (Portela et al. 2023; Aslam et al. 2024; Zié et al. 2025).

Ribeiro‐Filho et al. (2021) applied semi‐solid‐state fermentation (SSSF) with *Saccharomyces cerevisiae* to enrich the protein content of cashew apple bagasse, identifying parameters that render this matrix a viable alternative source of protein and dietary fiber. In that study, the most favorable fermentation conditions were identified as a yeast concentration of 12% (w/w), a temperature of 33°C, and an incubation period of 24 h. Cashew apple bagasse has considerable nutritional potential. Dried cashew apple bagasse has been reported to contain 9.9% protein and 35.0% total dietary fiber, comprising 8.0% soluble fiber and 27.0% insoluble fiber (Zié et al. 2023). Fermentation also promotes a more complex aromatic profile, broadening the ingredient's applicability in food formulations. In this regard, the research group responsible for the present study has investigated the incorporation of fermented, protein‐enriched cashew bagasse flour into plant‐based products, obtaining favorable results in physicochemical and structural attributes. However, sensory analyses linked to consumers’ emotional responses remain underexplored for this product category.

Acceptance of plant‐based meat analogs is influenced by product‐related factors, including perceived sensory attributes, nutritional value, and price (Onwezen et al. 2021). For consumers with limited exposure to this category, unfamiliarity and the perception of novelty represent a primary barrier, generating emotional discomfort, particularly among adults with well‐established taste references (Souza Olegario 2021; Onwezen et al. 2022). This discomfort is more pronounced when products resemble traditional foods, creating expectations that may not be met. Sensory and affective memory can thus function both as a bridge and as a barrier to acceptance (Eckl et al. 2021).

Beyond acceptability, food‐evoked emotions influence food choice and may predict consumption behavior more effectively than acceptance ratings alone (Tuorila and Hartmann 2020). In a longitudinal online survey with Dutch consumers, Onwezen et al. (2022) assessed anticipated emotions and consumption intentions toward fish, seaweed, insects, pulses, and cultured meat, without product tasting. Positive anticipated emotions, such as “happy,” “satisfied,” and “proud,” were identified as relevant determinants of the intention to consume alternative proteins, reinforcing the importance of measuring emotional responses to better understand acceptance in this product category.

Although various agro‐industrial co‐products have been investigated as ingredients in plant‐based and hybrid meat products, information regarding the use of fermented cashew apple peduncle bagasse enriched with microbial protein remains limited. Compared to the direct incorporation of conventional agro‐industrial residues, fermentation and yeast enrichment can enhance the nutritional and technological potential of this co‐product. However, its application in meat analogs and hybrids remains underexplored, particularly concerning sensory aspects.

Based on this knowledge gap, it was hypothesized that partially replacing soy protein or beef with cashew apple bagasse flour enriched through SSSF would produce concentration and matrix‐dependent changes in the characteristics of the products. Therefore, the present study evaluated the impact of partially replacing soy protein or beef with cashew apple bagasse flour enriched through SSSF on the instrumental properties, sensory profile, and consumer‐evoked emotional responses in plant‐based and hybrid meatballs.

## 2 Material and Methods

### 2.1 Materials

Cashew apple bagasse was supplied by a fruit pulp processing company located in the region of João Pessoa. The yeast *S. cerevisiae* (Fleischmann Royal) was obtained from a local distributor of baking ingredients.

### 2.2 Production of Protein‐Enriched Cashew Bagasse Flour

Protein‐enriched cashew bagasse flour was produced via SSSF following the optimized parameters described by Ribeiro‐Filho et al. (2021). Initially, the bagasse was spread onto aluminum trays and homogenized with cashew juice at 2% (w/w). The material was then inoculated with *S. cerevisiae* (Fleischmann Royal) at a concentration of 12% (w/w). The yeast was previously activated by dissolving 1.8 g of dry yeast into 10 mL of water at 35°C. After inoculation, the mass was fermented in a forced‐air drying oven (air velocity 1 m/s; SOLAB SL‐102) at 33°C for 24 h. At the end of fermentation, the temperature was increased to 60°C and the fermented bagasse was dried for an additional 24 h. The dried material was ground in a food processor and sieved through a 25‐mesh screen. The enriched flour was packaged in plastic bags and stored at −18°C until analysis.

### 2.3 Preparation of Plant‐Based and Hybrid Meatballs

Plant‐based and hybrid meatballs were prepared in two independent batches, totaling 70 experimental units per treatment, according to the method described by Lima et al. (2017), with modifications. Four treatments were produced for each product type, varying the replacement level of soy protein or beef at 0%, 10%, 20%, and 30%.

For plant‐based meatballs, textured soy protein was hydrated at a 1:2 (w/w) ratio with water at 37°C for 15 min before ingredient incorporation. Adjustments were made to the added vegetable fat content (an increase of 1.3% for each 10% substitution with enriched flour). For hybrid products, ground beef (chuck cut) served as the base and was mixed with the remaining ingredients. The proportions of all components used in both formulations are presented in Table S1. The homogenized mixtures were shaped into characteristic meatball forms, each weighing 15 g. All meatballs were packaged in expanded polystyrene (EPS) trays, wrapped with plastic film, and stored at −18°C until analysis and sensory.

### 2.4 Instrumental Color Measurement

Colorimetric analysis was performed by measuring L* (lightness, black–white), a* (green–red), and b* (blue–yellow) parameters using a portable colorimeter (Konica Minolta, model CR‐10), following the CIELAB system (Jaramillo‐Sánchez et al. 2018).

### 2.5 Texture Profile Analysis

Texture profile analysis (TPA) was conducted using a TA.XT Plus texture analyzer (Stable Micro Systems, Surrey, UK), following an adapted methodology from Samutsri et al. (2023). Samples were baked at 180°C for 10 min and compressed twice using a P/2 probe with a trigger force of 5 g (0.049 N). Pre‐test, test, and post‐test speeds were set at 2 mm/s, with a compression distance of 12 mm. Five replicates per treatment were analyzed for hardness (N), gumminess (N), chewiness (N), springiness, cohesiveness, and resilience.

### 2.6 Volatile Compound Profiling

Volatile organic compounds (VOCs) were analyzed in the samples with the highest sensory acceptance (plant‐based analog containing 20% enriched flour and hybrid containing 10%, along with their respective controls). Formulations containing 30% enriched flour were excluded from this stage due to inadequate sensory and instrumental performance. VOC determination was conducted using headspace solid‐phase microextraction (HS‐SPME) coupled with gas chromatography–mass spectrometry (GC–MS), following the method described by Hu et al. (2025), with modifications.

Briefly, 10 g of sample were homogenized with 10 mL of distilled water in 50 mL vials and equilibrated for 20 min at 60°C. Extraction was performed using a 75 µm Black SPME fiber, previously conditioned at 300°C for 1 h, exposed to the sample headspace for 40 min. As an internal standard, 3 µL of 1,2‐dichlorobenzene were added.

After extraction, volatile compounds were desorbed in the GC injector at 200°C. Analyses were performed using a GC–MS system equipped with an Agilent DB‐WAX capillary column (30 m × 0.25 mm ID × 0.2 µm film thickness). Helium was used as the carrier gas at a constant flow rate of 1.0 mL/min. The oven temperature program began at 40°C (held for 5 min), increased to 50°C at 8°C/min, then to 120°C at 10°C/min (held for 10 min), and finally increased to 240°C at 10°C/min and held for 5 min. The detector temperature was set at 250°C. Eluted volatile compounds were identified by comparing mass spectra with the NIST library and, when necessary, by calculating retention indices (RI) using a standard alkane series and comparing them with literature values.

### 2.7 Sensory Analysis

#### 2.7.1 Participants

This study was approved by the Ethics Committee of the Federal University of Paraiba (Universidade Federal da Paraíba—CAEE 91437525.5.0000.5188). Untrained adult participants were recruited for the Free Choice Profiling (FCP) tests (*n* = 24—hybrid; *n* = 26—analog; some participants took part in both FCP panels) and the Preferred Attribute Elicitation (PAE) test (*n* = 20; independent panel, with no overlap with the FCP participants), with mixed gender distribution. Recruitment criteria included: (i) no aversion to any ingredient used in the formulations, (ii) legal adulthood, and (iii) absence of soy sensitivity or adverse reactions. For the evaluation of evoked emotions (*n* = 100; group recruited from the general public), the only recruitment criterion was legal adulthood. Additional information regarding the profile of the described participants is presented in Table S8.

#### 2.7.2 Preference Order Test and Purchase Intent

Preliminary tests were conducted to identify treatments with the greatest acceptance potential and exclude less attractive formulations using a preference order test (POT) and a purchase intent (PI) test. The POT test identified relative preference among formulations without estimating preference intensity. The PI test, included on the same evaluation form, complemented this assessment using a 5‐point scale ranging from 5 (“certainly would buy”) to 1 (“certainly would not buy”) (Figures S1 and S2) (de Lima et al. 2020).

#### 2.7.3 Free Choice Profiling

In the FCP methodology, participants freely defined their own descriptive attributes. Prior to evaluation, participants received instructions on completing the sensory form and conducting individual assessments. Four treatments (0%, 10%, 20%, and 30% incorporation of enriched flour) were evaluated to identify descriptors associated with each treatment, define product profiles, and support selection and exclusion criteria. Samples were placed on EPS trays, coded with three‐digit numbers, and presented clockwise according to a completely randomized block design. The test was conducted in individual booths under white lighting, adequate ventilation, and noise‐free conditions. Sessions for analog and hybrid products were conducted on separate days to prevent sensory fatigue and direct comparison between categories. Participants indicated perceived intensity using a 10‐cm unstructured scale following Liu et al. (2018), with adaptations.

#### 2.7.4 Preferred Attribute Elicitation

The PAE procedure for both product categories (plant‐based and hybrid) followed the methodology described by da Silva et al. (2025). Treatments were defined based on preliminary sensory tests (POT, PI, and FCP) and instrumental data, which indicated exclusion of the 30% enriched flour formulation. Thus, samples containing 0%, 10%, and 20% enriched flour were evaluated for both product categories.

On the day before the sensory evaluation, the samples were transferred from the ultra‐low‐temperature freezer to a conventional freezer, where they remained frozen until preparation. On the day of the evaluation, the samples were cooked directly from the frozen state in an air fryer at 180°C for 10 min. Approximately 8 min after cooking, while still warm, all samples were simultaneously presented to the panelists in randomized order. The samples were served in white plastic cups coded with three‐digit numbers. Water and plain crackers were provided for palate cleansing. The same storage, cooking, and post‐cooking conditions were applied to the samples used for instrumental texture analysis.

In the first stage, the acceptability of each treatment was evaluated individually. Next, participants indicated attributes that characterized the products. The listed terms were discussed as a group to establish consensus on the most representative descriptors. A total of 19 attributes were selected and organized into appearance, aroma, flavor, and texture categories, and participants’ understanding of the 9‐point scale was confirmed. After a 15‐min interval, participants individually evaluated the intensity of each attribute for each sample. Finally, they ranked attributes from most to least important. The session lasted approximately 120 min.

#### 2.7.5 Evaluation of Evoked Emotions

The evaluation of evoked emotions followed the methodology described by Jaeger et al. (2019), with modifications. Thirty‐three facial emojis validated for food‐related applications (da Cruz et al. 2021) were used and presented in randomized order alongside the stimulus image (Figure S3).

Two questionnaires were developed, one for the plant‐based analog and another for the hybrid product, each structured in two stages: the first presenting an image of a traditional product (plant‐based or hybrid), and the second presenting the product incorporating enriched cashew bagasse flour. There was no involvement of the palate at this stage, only visual stimuli (images) were used.

Participants were instructed as follows: *“Based on the image shown, select the emojis that best represent the emotions triggered by the proposed product (select all that apply).”*

### 2.8 Data Analysis

Statistical analyses of sensory and emotional data were performed using XLSTAT 2022.2.1 (Addinsoft, New York, USA). Collected data were processed using PAST version 4.03 (PAleontological STatistics; Hammer et al. 2001), software developed by Øyvind Hammer, University of Oslo, Oslo, Norway. Results were subjected to analysis of variance (ANOVA) at a 5% significance level followed by Tukey's test for proximate composition, texture profile, and color parameters. The volatile compound profile was explored using heat maps generated in R software (version R 3.6.0) to visualize similarity patterns among treatments and identified compounds.

PAE data were analyzed using generalized procrustes analysis (GPA), based on a matrix with 6 rows (formulations) and 380 columns (19 attributes × 20 consumers). Attribute importance was assessed using the Friedman test (*p* < 0.05). Sensory acceptance was evaluated using one‐way ANOVA and Tukey's test (*p* < 0.05). FCP data were also analyzed using GPA.

For emotional response analysis, Cochran's *Q* test combined with McNemar's test (Bonferroni‐adjusted) was used to evaluate differences in emoji citation frequencies among formulations using CATA data. A global chi‐square test assessed independence between rows and columns in the contingency table, and cell chi‐square values were used to identify contributions to overall variation. In addition, a correspondence analysis (CA) map was constructed using a matrix of 400 rows (100 consumers × 4 formulations) and 33 columns (emojis).

## 3 Results and Discussion

### 3.1 Meatball Characterization

**FIGURE 1:**
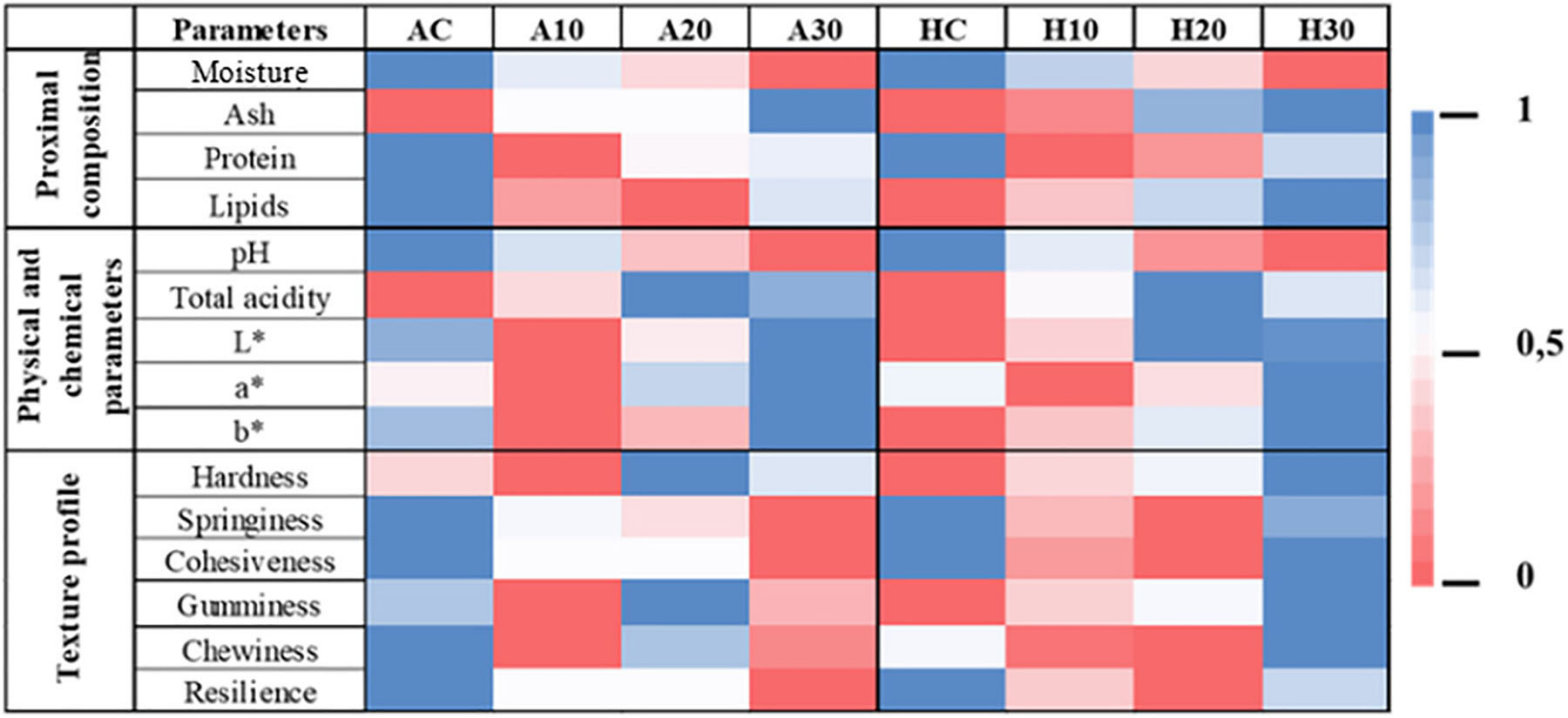
Heatmap of proximate composition, instrumental color parameters, and texture profile of analog and hybrid meatballs. The color scale was applied independently to each analytical parameter using conditional formatting in Microsoft Excel. Within each row, the color gradient represents the relative variation among formulations, ranging from the lowest to the highest observed value. Therefore, color intensities should be interpreted within each parameter and should not be compared directly across proximate composition, instrumental color, and texture variables.

The results of the proximate composition, physicochemical parameters, instrumental color, and texture analysis of plant‐based and hybrid meatballs are shown in Figure 1. Regarding the proximate composition, moisture content decreased significantly with the incorporation of enriched flour in both product categories (*p* < 0.05) (Table S2). In analog meatballs, moisture decreased from 62.67% in the control formulation (AC) to 57.51%, 55.72%, and 52.92% in A10, A20, and A30, respectively. A10 and A20 did not differ significantly from each other (*p* > 0.05), whereas the other comparisons were significant (*p* < 0.05). In hybrid meatballs, moisture decreased from 62.48% in HC to 50.44% in H30, with significant differences among all formulations (*p* < 0.05).

However, no change in the protein content was observed among the samples for each category, that is, the incorporation of enriched cashew meal maintained the protein content of the plant‐based and hybrid samples, even though soy and beef protein content of the formulations was lower due to the substitution. A study by Nam et al. (2024) reported a significant increase in the protein content of plant‐based patties supplemented with biji powder, a fiber‐ and protein‐containing soybean by‐product. Substitutions of the meat by the enriched flour caused an increase in total ash percentage of the hybrid meatballs for formulations with 20% and 30%, while no difference was observed between the plant‐based samples. The incorporation of enriched flour decreased the lipid content of the plant‐based meatballs, from 6.31% in AC to 2.73%, 2.38%, and 3.94% in A10, A20, and A30, respectively (*p* < 0.05), and increased it in the hybrid samples from 3.53% in HC to 5.12%, 6.97%, and 8.83% in H10, H20, and H30, respectively.

Muscle fibers retain water through myofibrillar binding, whereas textured soy proteins retain water according to their structural and rehydration properties, which are influenced by characteristics such as density and porosity (Van Esbroeck et al. 2024). In this context, the incorporation of protein‐enriched cashew apple bagasse flour may result in a less pronounced reduction in moisture content in the plant‐based matrix than in the hybrid matrix, possibly due to the water absorption and retention characteristics of the textured soy protein matrix. This difference suggests that the interaction of the flour with each protein matrix may have contributed to the distinct reductions in moisture content. Lin and Barbut (2025) demonstrated that the type and structural characteristics of soy proteins influenced water mobility and cooking loss in hybrid meat systems.

Regarding lipid behavior, divergent patterns were observed between hybrid and analog products. In soy‐based systems, lipids are rapidly absorbed but less effectively retained over time. In hybrid formulations, the interaction between meat proteins and cashew bagasse fibers forms a more cohesive matrix capable of better retaining the added vegetable oil, resulting in a more stable and higher lipid content than in analog products. This highlights distinct interactions within each matrix (Santos et al. 2022).

pH values decreased significantly with increasing concentrations of the fermented ingredient within both product categories (Table S2). In analog meatballs, the pH decreased from 6.30 in the control formulation to 6.01, 5.83, and 5.69 in A10, A20, and A30, respectively, with significant differences among all analog formulations (*p* < 0.05). In hybrid meatballs, the pH decreased from 5.92 in the control to 5.63, 5.53, and 5.51 in H10, H20, and H30, respectively. The hybrid control differed significantly from all flour‐containing formulations (*p* < 0.05). No significant differences were observed between H10 and H20 or between H20 and H30 (*p* > 0.05), whereas H10 and H30 differed significantly from each other (*p* < 0.05). This reduction was more pronounced in analog products than in hybrids, possibly because nitrogenous compounds in the meat matrix provide buffering capacity and attenuate pH variation.

### 3.2 Instrumental Color Determination

For the plant‐based analog treatments, the L* parameter (lightness), shown in Figure 1, remained constant. For the a* parameter (red–green), a significant difference was observed at the 10% incorporation level, whereas the control, 20%, and 30% treatments did not differ statistically (*p* < 0,05; Table S3); at higher incorporation levels (20%–30%), the reddish tone stabilized. For the b* parameter (yellow–blue), the control, 20%, and 30% treatments were statistically similar, while the 10% treatment differed from the others, with the 20% level exhibiting an intermediate behavior.

The observed color differences may have been influenced by the distinct interactions between the enriched cashew apple bagasse flour and the matrix (textured soy protein in the analog products and the meat in the hybrid products), during baking. These interactions may have altered matrix structure and density, potentially affecting heat transfer. Surface browning, possibly associated with Maillard reactions, may also have contributed to the instrumental color variations, since the extent and products of these reactions depend on the type and availability of amino compounds and reducing sugars present in the food matrix (Shakoor et al. 2022). However, these complex mechanisms were not directly evaluated and warrant further investigation. Fermented cashew bagasse contains pigments and compounds that may either reduce or intensify yellow coloration depending on concentration, often imparting a more orange hue through effects on the b* (yellow) parameter. At 10% incorporation, pigment levels are insufficient to stabilize color, yet sufficient to modify b* relative to the control, thereby affecting the final visual tone. In plant‐protein‐based and hybrid products, final color depends on both Maillard reactions and the natural pigments of the ingredients. Deen et al. (2025) attributed color changes in fermented plant materials to enzymatic activity during fermentation, which can alter pigment compounds.

For hybrid meatballs, flour incorporation resulted in a lighter surface (L*: 26.08 ± 0.52 in the control, increasing to 30.85 ± 0.92 at 30% incorporation; Table S3). Reduced moisture associated with fiber addition to a meat matrix during heating can lead to either increased or decreased browning (Słowiński et al. 2021). In addition to reducing water retention, bagasse alters product density and structure, facilitating more uniform heat transfer and influencing color development (Hong et al. 2022).

Regarding the a* parameter, only the 30% formulation showed a significantly higher value (approximately +4), while the others ranged from +2.15 to +2.75. For the b* parameter, no significant differences were observed among formulations. Due to Maillard reactions and pigments inherent to meat, coloration tends toward brown tones, driven by the combined influence of a* (redness) and lightness. This stability pattern was similar to that reported for soy‐based fermented analog products (Deen et al. 2025).

### 3.3 Texture Profile

Texture profile data (Figure 1 and Table S4) indicate that, in the analog samples, no significant differences were observed for hardness or gumminess. In contrast, hybrid formulations exhibited higher values, with the 30% incorporation level being significantly greater than the other treatments. These mechanical properties directly influenced chewiness, which was also higher in the hybrid products. Chewiness is associated with flavor release and reflects resistance to deformation during mastication, thereby influencing sensory perception and helping explain the lower perceived seasoning intensity at higher incorporation levels (e.g., 30%), which were described as less “seasoned.” Similar behavior in plant‐based products has been reported by Rasul et al. (2025), who linked firmer textures to reduced flavor release.

Regarding elasticity, analog samples showed significant reductions compared with the control (*p* < 0.05), with no further significant differences as the incorporation level increased. In contrast, hybrid samples exhibited a non‐linear response: formulations containing 10% and 20% enriched flour showed significant reductions compared with the control (*p* < 0.05), whereas the 30% formulation showed an increase in elasticity and did not differ significantly from the control (*p* > 0.05). This effect is associated with reorganization of the myofibrillar protein network at higher fiber levels, contributing to a more cohesive and resistant structure and partially restoring elasticity (Dunne et al. 2025; Beldarrain et al. 2020). For cohesiveness, a reduction was observed with increasing bagasse incorporation, particularly at higher concentrations in analog formulations (A30: 0.26 ± 0.04), while no significant variation was observed in hybrid products.

Recovery after deformation was limited in analog products because the more fibrous and less cohesive plant matrix does not efficiently retain its structure, due to weaker internal bonding forces, reducing the product's ability to withstand a second deformation. The greater resilience observed in hybrid products reflects the presence of the meat matrix, which provides enhanced structural support. In analogs, the lower density of internal bonding forces reduces the capacity to withstand repeated deformation. In hybrids, cohesiveness remained more stable with bagasse addition because the meat matrix provides greater structural integrity (Serdaroğlu et al. 2018).

### 3.4 Volatile Compounds in Analog and Hybrid Meatballs

A total of 74 volatile compounds were identified in the analog and hybrid meatballs (Figure 5) and grouped into chemical families as follows: aldehydes (9), alcohols (10), alkanes/hydrocarbons (2), ketones (3), esters (5), acids and derivatives (3), nitrogen‐containing compounds (1), aromatic phenolics (4), and sulfur‐containing compounds (9). In addition, terpene compounds were identified, represented by monoterpenes (12) and sesquiterpenes (16), which contributed significantly to the aromatic complexity of the formulations. The most abundant groups—terpenes and sesquiterpenes, followed by alcohols and aldehydes—potentially impacted sensory attributes such as fresh, fruity, and spicy aromas, as well as characteristics associated with processed meat products.

In the analog meatballs, particularly AC (conventional), a higher relative abundance of sulfur‐containing and terpene compounds was observed, such as diallyl sulfide and methyl allyl disulfide. These compounds have a sensory impact and are associated with intense garlic and onion notes, as well as cooked seasoning nuances (Kilic‐Buyukkurt et al. 2023; Wang et al. 2023). This result is consistent with the “seasoned” descriptor identified in the sensory analysis. In addition, a contribution of terpenes and sesquiterpenes (alloaromadendrene, δ‐elemene, humulene, γ‐terpinene, α‐cubebene, sabinene, D‐limonene, γ‐muurolene, terpinen‐4‐ol, α‐muurolene, β‐bisabolene, cis‐calamenene, α‐terpinene, β‐elemene, β‐citral, and β‐selinene) was observed. Terpenoids are important aroma components in vegetable matrices and may contribute to herbal, green, and citrus notes that are characteristic signatures of plant‐based matrices, reinforcing the plant‐based character (Tian et al. 2024).

Conversely, in HC, the predominant profile consisted of aldehydes such as pentanal, butanal, and 3‐methyl, compounds commonly associated with meat products and responsible for lipid‐derived and “fatty” aroma notes. Aromatic descriptors such as “fatty,” also reported by Li et al. (2022) in fermented sausage products, highlight the similarity between the identified aromatic profile and those characteristic of meat matrices.

On the other hand, the incorporation of fermented cashew bagasse into both matrices modulated distinct aromatic profiles. The A20 formulation, containing 20% enriched cashew bagasse flour, exhibited an aromatic profile marked by the presence of pyrazine, 2‐ethyl‐6‐methyl, a compound typical of Maillard reactions and associated with nutty and roasted descriptors. In addition, phenolic compounds such as phenol, 4‐ethyl‐2‐methoxy (4‐ethylguaiacol) and the monoterpene p‐cymene suggested smoky, spicy, and woody characteristics (Tian et al. 2024). Reinforcing the aromatic complexity of this sample and contributing to a more attractive and seasoned profile favorable for improving plant‐based product characteristics. In general, the “fermented” character was more intense compared to the control samples and may dominate the product aroma, masking and/or reducing the relative contribution of terpene notes associated with seasonings observed in the control, a response corroborated by the sensory descriptive profiles obtained in the present study.

Finally, in the hybrid treatment (H10), the presence of fermented cashew bagasse was associated with sulfur compounds (diallyl sulfide and methyl allyl disulfide), favoring seasoned and fermentative aromatic nuances, as well as esters that added aromatic complexity. In hybrid formulations, a more balanced profile was observed, without an exclusive predominance of fermentation‐derived volatiles, highlighting the presence of sesquiterpenes such as β‐cis‐caryophyllene, which may contribute spicy notes, as reported in chicken–spice systems (Andaleeb et al. 2024). However, in the incorporation treatment, the presence of esters such as ethyl isobutyrate and ethyl isovalerate added subtle aromatic complexity, introducing sweet fruity notes derived from the bagasse and its interaction with the hybrid matrix. This aromatic complexity introduced by the fermented plant matrix results in a sensory profile less aligned with consumer expectations for meat products. At higher concentrations, it reduced acceptance, as evidenced in the sensory tests described in the present study.

It is important to emphasize that not all intensified volatiles are necessarily positive, depending on concentration. Aldehydes such as hexanal, although contributing meat‐like and fatty notes at low levels, generate rancid or oxidized perceptions when present in excess. Likewise, fermentative and acidic compounds intensify vinegary or alcoholic notes when overly pronounced, which may negatively impact acceptance depending on the sensory balance. Thus, the main advantage of fermented bagasse was promoting a more complex and distinctive aroma, with an increase in fruity, seasoned, and meat‐associated compounds in products containing fermented cashew bagasse flour, reinforcing its potential as a functional ingredient.

### 3.5 Characterization of the Sensory Attributes of Analog and Hybrid Meatballs

#### 3.5.1 Free Choice Profile

**FIGURE 2:**
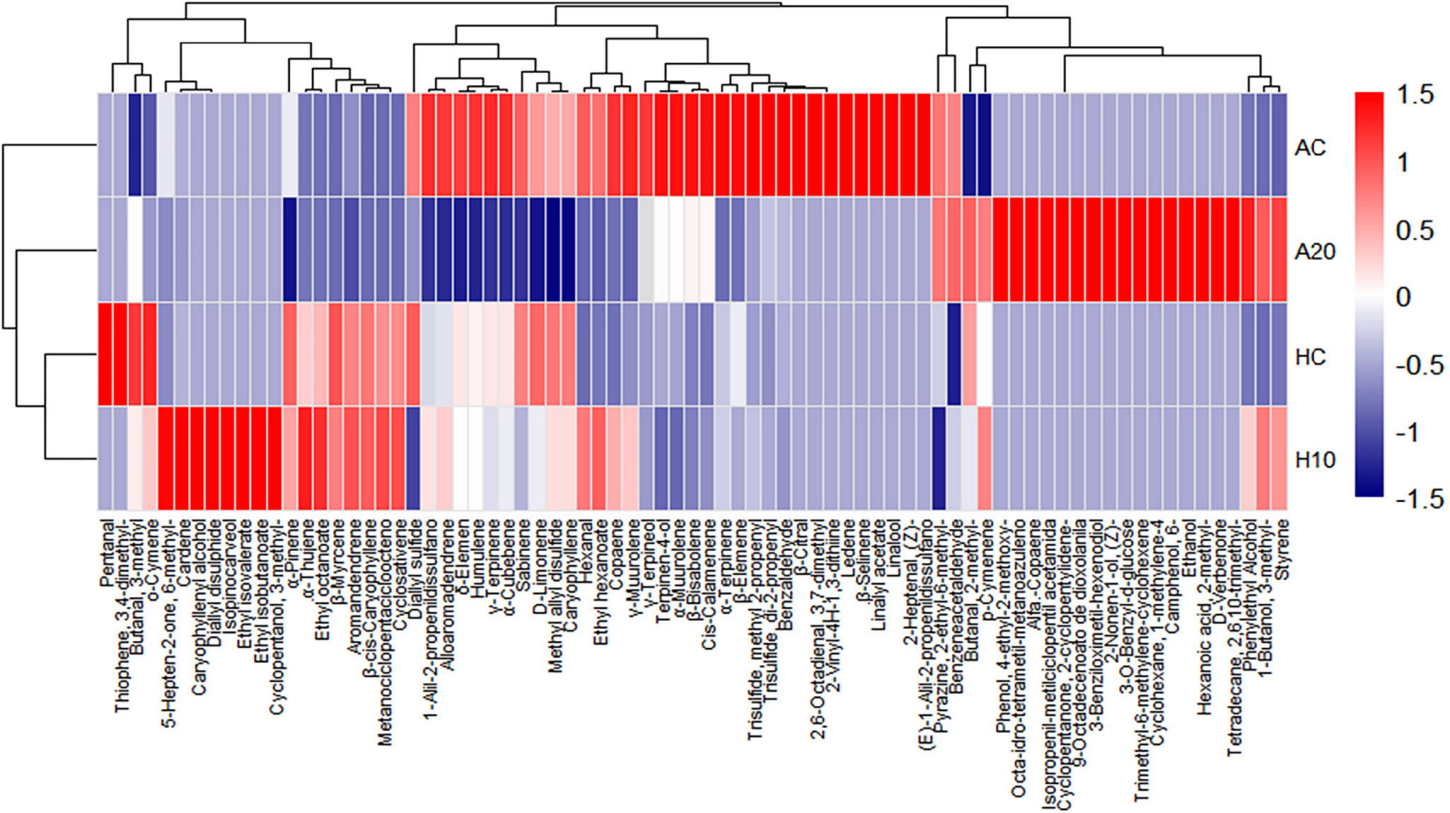
Heatmap of volatile compound profile in analog and hybrid meatballs formulated. Data were standardized for each volatile compound. Red and blue colors indicate relative values above and below the mean of each compound across the formulations, respectively, whereas white indicates values close to the mean. The dendrogram at the top represents the hierarchical clustering of volatile compounds based on similarities in their relative profiles across the formulations, while the dendrogram on the left represents the clustering of formulations according to their overall volatile profiles. The order of the compounds and formulations was determined automatically by hierarchical clustering.

GPA of the free choice profile data (Figure 2) revealed a broad dispersion of descriptive terms, reflecting the diversity of evaluators’ perceptions. The GPA configuration explained 89.6% of the variance for hybrid samples (*F*1 = 75.4%; *F*2 = 14.2%) and 84.0% for analog samples (*F*1 = 61.7%; *F*2 = 22.3%).

For hybrid formulations, the primary component (horizontal axis; Figure 2A,B) separated samples with lower incorporation levels (HC and H10) from those with higher levels (H20 and H30), indicating the influence of enriched cashew bagasse flour concentration on sensory differentiation. The second component (vertical axis; Figure 2C,D) distinguished H10 and H30 from HC and H20, capturing qualitative variations in attributes such as texture, appearance, and flavor that depend not only on bagasse concentration but also on how it modifies the perception of specific characteristics.

Along the primary axis (F1), attributes associated with HC and H10 were characteristic of meat‐based products, including meaty, roasted color, tender, and seasoned. These descriptors suggest an indirect influence of texture on flavor perception, reinforcing sensory cues typical of traditional meat profiles. In contrast, H20 and H30 were associated with descriptors such as cashew, fermented, and uniform, reflecting more pronounced divergences and perceptual changes linked to incorporation of the plant‐based matrix.

On the secondary axis (F2), HC and H20 were associated with textural attributes (juicy, tender, elastic, dry), whereas H10 and H30 were linked to flavor and appearance descriptors (seasoned, spiced, attractive, dry, cashew). The descriptor fermented emerged as a relevant marker of formulations containing higher levels of enriched cashew bagasse flour. Fermentation processes, or the incorporation of fermented matrices into food products, enhance volatile compound profiles and modify texture parameters, thereby influencing flavor perception (Li et al. 2022), as observed in the sensory results.

For the analog GPA, the primary component separated formulations according to bagasse content. AC and A10 clustered on the left and were associated with descriptors such as soy, seasoned, juicy, spiced, meaty, and uniform. In contrast, A20 and A30 clustered on the right and were associated with cashew, roasted color, attractive, fermented, grainy, and acidic. These distinctions reflect differences perceived by panelists and corroborated by instrumental data regarding similarities, differences, and the attributes most characteristic of each treatment. Samples with higher incorporation levels were primarily characterized by the fermented note, which partially masked the presence of soy despite its predominance in the formulation, a characteristic typically associated with plant‐based products.

On the second component, separation occurred along the vertical axis, with samples positioned in the upper region (AC, A20, and A30) associated with texture‐impacting attributes such as dry, fibrous, peppery, and attractive, while the lower region (A10) was associated with smoky, juicy, toasted, tender, and soy. These results indicate that low levels of bagasse incorporation (A10) preserve characteristics close to the control while introducing subtle flavor and texture nuances. In contrast, higher incorporation levels (A20 and A30) intensified descriptors related to cashew, fermented notes, and grainy texture, highlighting the ingredient's influence on the sensory profile and overall product perception.

Recent work by Heo et al. (2023), evaluated 10 commercial bottled waters and one filtered water using descriptive analysis, FCP, and polarized sensory positioning, demonstrated that sensory profiles generated through free descriptors can effectively discriminate sample groups based on dominant sensory attributes, even when differences are subtle, as observed in the present study.

#### 3.5.2 Preferred Attribute Elicitation

**FIGURE 3:**
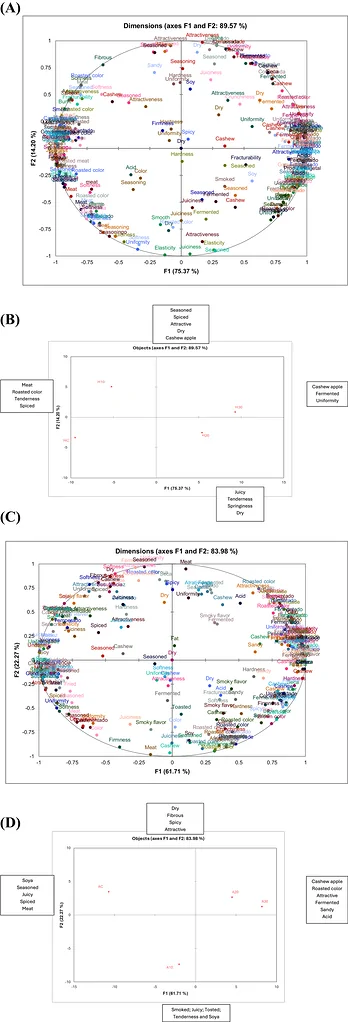
(A) Correlation of the GPA analysis of the free‐choice profile data for hybrid meatballs. (B) Two‐dimensional GPA graph of the differences between hybrid meatballs. (C) Correlation of the GPA analysis of the free‐choice profile data for analog meatballs. (D) Two‐dimensional GPA graph of the differences analog meatballs.

For the PAE tests, formulations with 30% substitution were excluded. Based on the 9‐point hedonic scale, scores for analog products (Figure 3; Table S5) remained stable, indicating tolerance for enriched flour incorporation up to 20% without significant loss of acceptability across evaluated parameters. Hybrid products showed a divergent pattern, consistent with findings by Stanišić et al. (2025). This result is favorable given the environmental limitations associated with soybean monoculture and the fact that substitution did not significantly alter protein content.

The added plant matrix did not influence appearDance acceptability in either hybrid or analog products (Figure 3A). For aroma (Figure 3B), differences were more pronounced in hybrid samples, with acceptability decreasing as substitution levels increased. For texture and flavor (Figure 3C,D), no changes were observed in analogs, whereas hybrids showed a significant reduction at 20% substitution. These responses agree with Baune et al. (2023), who reported that acceptability of hybrid products tends to decline as the plant fraction increases, particularly for aroma and flavor, while appearance is less affected. Regarding texture, fiber addition is more perceptible in meat matrices, making structural changes more evident.

Treatments A20 and H10 stood out as the most promising formulations. Participants were able to perceive differences between the formulations, and their responses pointed to distinct optimal incorporation levels, also indicating promising sensory performance. This pattern may be related, at least in part, to the predominance of younger participants on the evaluation panel, who may be more receptive to changes and innovations. The variations in optimal concentrations also suggest that acceptability may be influenced by consumers' prior experiences and by the reference expectations established for each product category. This trend contrasts with findings by Grasso et al. (2022), who evaluated commercially available burgers made of 100% beef, 100% plant‐based ingredients, and a hybrid version containing 60% beef and 40% plant‐based ingredients. In blind tests, the hybrid burger was the only sample to achieve a mean overall acceptance score above 6 on a 9‐point hedonic scale, corresponding to “liked slightly,” and received higher ratings than the all‐plant‐based burger.

During the descriptive stage, panelists identified 19 attributes considered important for meatball‐type products. Desired characteristics included brown color, meaty aroma, seasoned flavor, and juicy texture, while undesirable attributes included dry appearance, fermented aroma, rancidity, fermented cashew flavor, and gumminess (Table S6). These responses highlight the role of reference expectations: identifying fermented cashew flavor as less important does not necessarily imply rejection but rather reflects that this characteristic is not expected in this product category.

**FIGURE 4:**
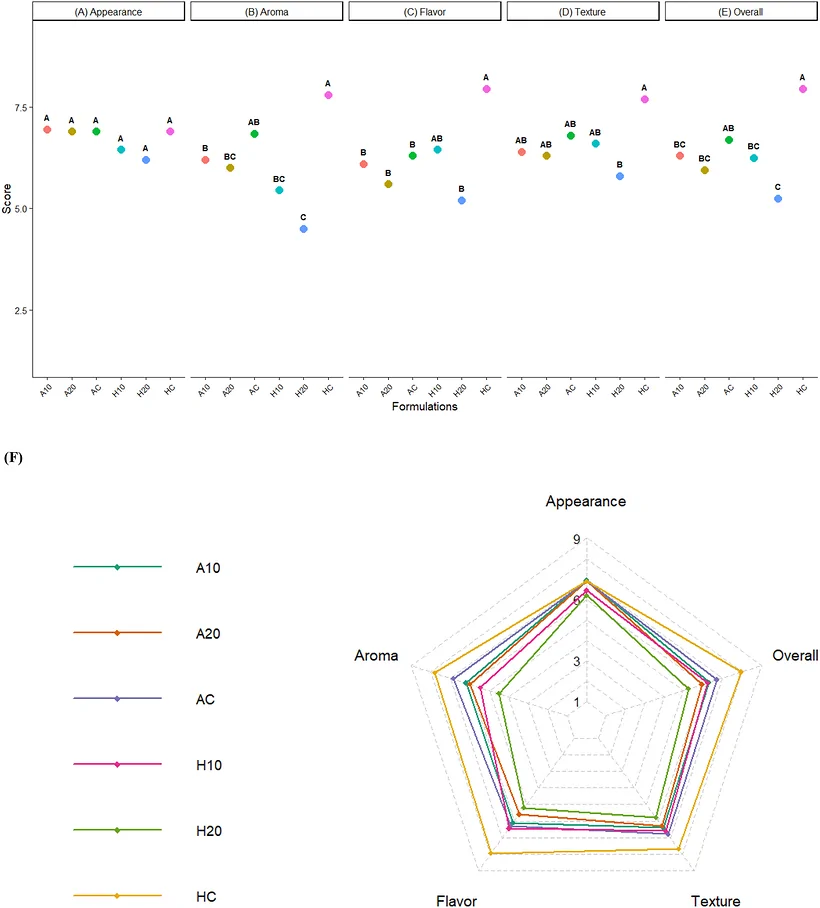
Sensory acceptability assessment of analog and hybrid meatballs with individual response trends for attributes of (A) appearance (B) aroma (C) flavor (D) texture (E) overall acceptance, and radar graph (spider plot) showing comparative overview of the formulations (F). AC: control analog meatball; A10: analog meatball prepared with 10% substitution of soy protein with enriched cashew apple flour; A20: analog meatball prepared with 20% substitution of soy protein with enriched cashew apple flour; HC: control hybrid meatball, H10: hybrid meatball prepared with 10% substitution of beef with enriched cashew apple flour; H20: hybrid meatball prepared with 20% substitution of beef with enriched cashew apple flour. Different uppercase letters above the points indicate significant differences among formulations within the same sensory attribute according to Tukey's test (*p* < 0.05). Formulations sharing at least one letter do not differ significantly.

GPA analysis (Figure 4 ), explaining 71.1% of variance with two principal components (PC1 = 47.2%; PC2 = 23.9%), revealed clear differences among formulations. The primary component separated control samples (HC and AC) from those containing bagasse (A10, A20, H10, and H20). Control samples were associated with stronger luminosity (in analogs), seasoning, meaty character, juiciness, and spiced flavor. In contrast, samples containing bagasse in both matrices were associated with dry appearance, fermented notes, cashew aroma, firmness, gumminess, fibrous texture, fermented cashew flavor, and rancid notes.

**FIGURE 5:**
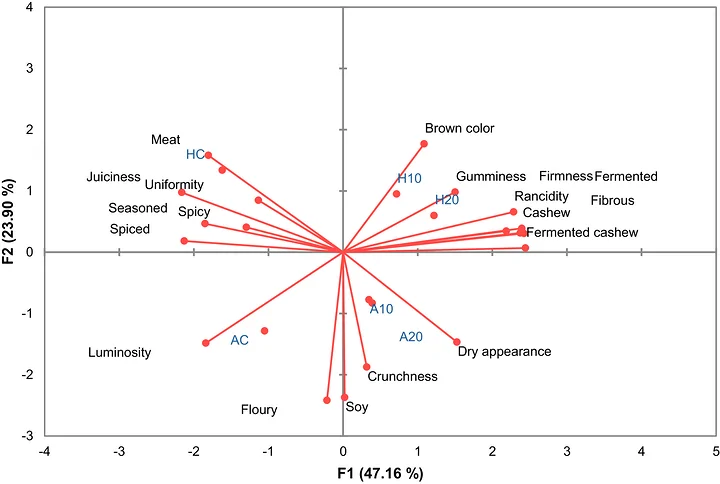
Principal component analysis (PCA) of 19 sensory attributes. AC: control analog meatball; A10: analog meatball prepared with 10% substitution of soy protein with enriched cashew apple flour; A20: analog meatball prepared with 20% substitution of soy protein with enriched cashew apple flour; HC: control hybrid meatball, H10: hybrid meatball prepared with 10% substitution of beef with enriched cashew apple flour; H20: hybrid meatball prepared with 20% substitution of beef with enriched cashew apple flour. The main descriptors correlated with the first two dimensions of the mean space are listed in the boxes.

In turn, along the second component (upper and lower axes), all hybrid formulations (HC, H10, H20) were positioned in the upper axis and were mainly associated with brown color, whereas the analog formulations (AC, A10, A20) were in the lower axis, associated with floury texture, crispness, and soy notes. Some attributes, such as uniformity and spiciness, showed no significant correlation, suggesting similar intensity across all samples. The analysis indicates that the incorporation of cashew bagasse primarily influences appearance and texture, while the distinction between hybrid and analog products is more evident in the second dimension, reflecting intrinsic matrix characteristics. Panelists also perceived fermented cashew aroma and flavor more intensely in the hybrid samples, whereas in the analog products, the most associated attributes were dry appearance and soy notes.

Popoola et al. (2019), using the PAE methodology for meat products, identified descriptors such as juiciness, beef/meaty aroma, tender texture, characteristic meaty flavor, and mild flavor and aroma, highlighting expected similarities in meat products or alternatives that shape sensory profiles and influence consumer acceptance.

#### 3.5.3 Evaluation of Evoked Emotions

**FIGURE 6:**
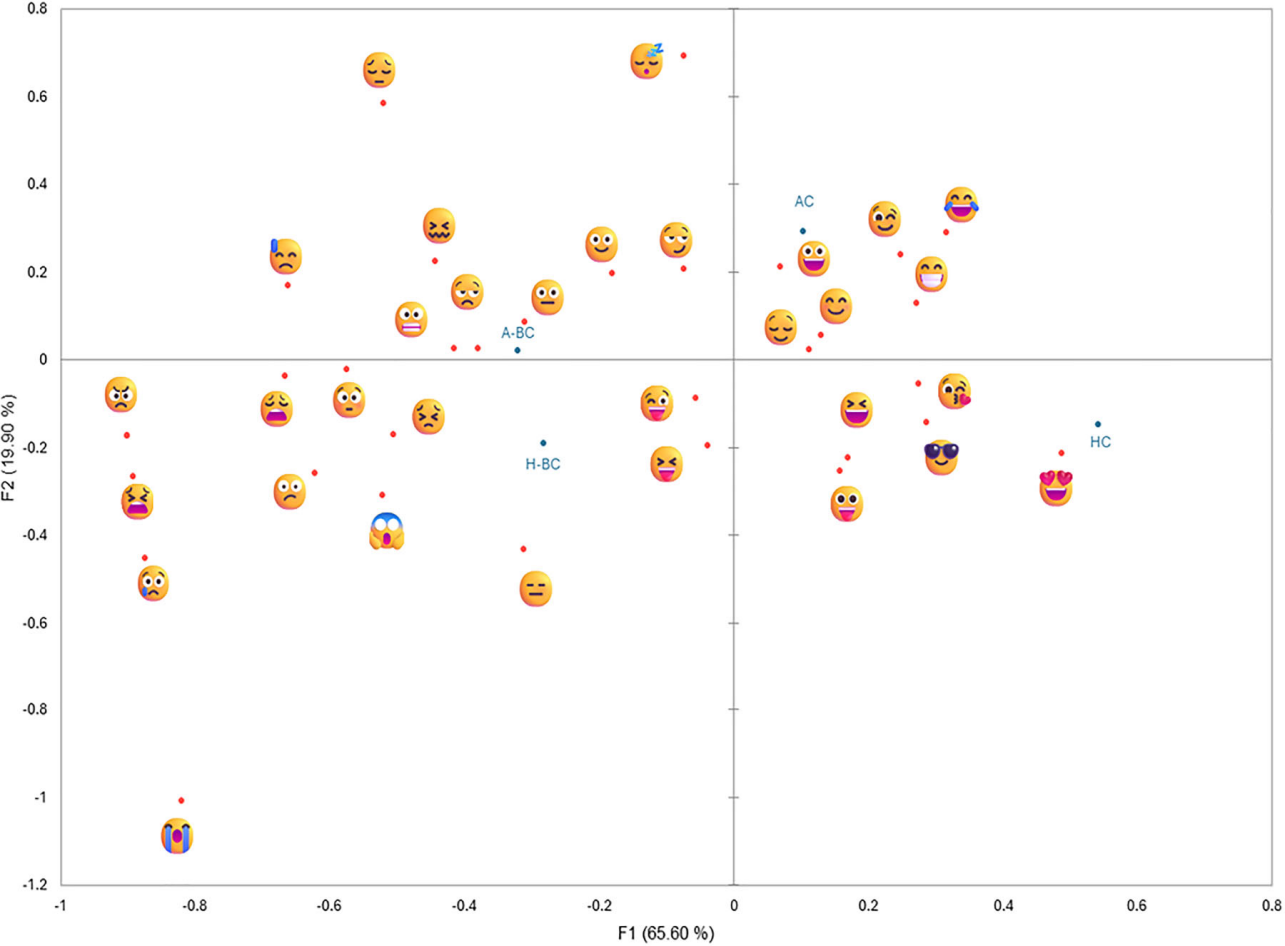
Principal component analysis (PCA) of evoked emoticons using emojis in analog and hybrid meatballs. AC: control analog meatball; A‐BC: analog meatball with substitution of soy protein with enriched cashew apple flour; HC: control hybrid meatball, H‐BC: hybrid meatball with substitution of beef with enriched cashew apple flour.

CA (Figure 6), explaining 85.1% of the variance with two PCs (PC1 = 65.6%, PC2 = 19.9%), revealed clear differences between conventional products and those containing bagasse incorporation. The principal components (left and right) separated conventional meat and analog products (HC and AC) from products incorporating fermented cashew bagasse (H‐BC and A‐BC), highlighting differences among treatments in emotional associations.

In the first component (left–right), there was a separation between conventional treatments and those containing fermented cashew bagasse. Emotional responses indicated a predominance of positive emotions for both conventional meat and analog products (HC—conventional meat; AC—conventional analog). However, when the incorporation of an agro‐industrial by‐product was proposed, emotions such as surprise and uncertainty were significantly evoked. Negative emojis were represented on the map for the incorporated analog and hybrid products (A‐BC and H‐BC), but they were not statistically significant (Table S7), thus not indicating rejection. The similar emotional patterns observed for both product categories, despite their distinct plant‐based and animal‐derived matrices, suggest that the responses were more closely associated with the novelty of cashew apple bagasse incorporation than with the type of product matrix. The most representative emoji was “surprised,” suggesting that the emotional profile reflects unfamiliarity and caution toward novelty rather than a truly negative reaction. Jakobsen et al. (2026) highlights how lack of familiarity with novel foods can generate initial apprehension.

The second component (upper–lower) separated analog from hybrid products, although less clearly than the first component. However, emojis associated with these formulations were not significant, with formulations located near the horizontal axis, indicating minimal emotional differences between analog and hybrid products. Therefore, what truly differentiates emotional perception among the proposed products is not the base matrix (plant or meat), but rather the reaction to the incorporation of fermented cashew bagasse. Consumer emotional profiles toward innovation depend on familiarity with the matrix and perception of novelty; this “familiarity” acts as a reference point but does not translate into rejection, rather into doubt, strangeness, and questioning. Previous studies have shown that unfamiliar and upcycled foods may evoke caution, uncertainty, or reluctance, with emotional responses and food neophobia influencing their acceptance (Tuorila and Hartmann 2020; Hellali and Koraï 2023). These responses reflect behavioral rigidity linked to preconceptions and psychological factors, impacting the introduction of new products to the general market, depending also on the target audience and how the product concept is presented.

Another perspective to consider is the age profile of the participants, with a predominance of the 18–25 and 26–35 age groups, this leads to the hypothesis that the sample's predominantly young composition may have influenced, at least in part, how the product concept was perceived. Younger people tend to be more open to innovation and change. In this context, even given a greater receptivity to innovation, this factor does not rule out feelings of surprise, caution, or uncertainty regarding an unexpected ingredient, such as cashew apple bagasse flour, yet it highlights the potential influence of age, considering that no emoji representing a negative emotion was significant, despite the lack of familiarity.

Overall, the results indicate that fermented cashew bagasse shows promising potential as a sustainable ingredient for application in analog and hybrid meat products, at distinct incorporation levels due to interactions with the base matrices. Fermentation contributed to technological and sensory improvements through modification of the fibrous matrix and development of aromatic compounds. Sensory and emotional analyses, integrated with instrumental parameters, enabled the establishment of a comparative profile of the ingredient in both matrices, highlighting impacts on volatiles, texture, color, sensory perception, and consumer affective responses. Agro‐industrial by‐products may also modulate emotions differently due to the novelty they introduce. Emotional aspects, even in the absence of taste involvement and instead driven by visual stimuli such as images, complement traditional sensory analysis, broadening the understanding of the consumer experience, as demonstrated in the present study.

Thus, fermented cashew bagasse stands out as a multifunctional ingredient, promoting technological quality, enhanced sensory profile, and sustainable innovation in plant‐based and hybrid products when incorporated at balanced concentrations.

Certain limitations of this study should be acknowledged. Among them, the participants' familiarity with plant‐based, hybrid, and upcycled foods was not directly assessed, which limits the interpretation of the extent to which prior experiences may have influenced their sensory and emotional responses. Furthermore, the mechanisms underlying matrix‐dependent changes, including protein–matrix interactions and specific reactions associated with color development, were not directly investigated.

However, the image‐based emotional assessment was an intentional methodological approach designed to evaluate participants' responses to the product concept prior to tasting; therefore, it should not be interpreted as a study limitation. Nevertheless, given the study's focus on consumer perception and sensory responses, the results offer relevant insights into the instrumental, sensory, and emotional effects of incorporating enriched cashew apple peduncle pomace flour into plant‐based and hybrid meatballs.

## 4 Conclusions

Overall, hybrid products showed greater sensitivity to incorporation in physicochemical and structural properties but exhibited a more balanced volatile profile. In contrast, analog formulations displayed distinct profiles, with the flour acting as a masking agent for terpenes typical of plant‐based products, possibly due to the perceived fermented character. Sensory changes detected instrumentally were perceived but did not compromise acceptability at balanced concentrations, with 10% (hybrids) and 20% (analogs) identified as the most promising levels. The PAE and FCP techniques identified 19 attributes and generated a broad perceptual map, indicating similarities among samples at different intensities. Emotional tests indicated greater apprehension toward the unfamiliar ingredient than toward the base matrix, with surprise and questioning rather than rejection. The integrated analysis of instrumental, sensory, and emotional methods allows understanding of consumer perception beyond product characteristics, highlighting the potential of enriched cashew bagasse flour as an innovative ingredient. However, its application requires balance and careful formulation, with further studies needed on processing strategies, by‐product combinations, and sensory alignment with consumer perceptions and emotions.

## Author Contributions

**Natália Ferreira Negreiros**: conceptualization, methodology, data curation, formal analysis, writing – original draft, writing – review and editing. **Rogério Silva de Almeida**: methodology, data curation, formal analysis. **Tatiana Colombo Pimentel**: methodology, data curation, formal analysis. **Mercia de Sousa Galvão**: methodology, data curation, formal analysis. **Tânia Beatriz Santos Cândido**: methodology, data curation, formal analysis. **Leila Moreira de Carvalho**: methodology, data curation, formal analysis. **Normando Mendes Ribeiro‐filho**: methodology, conceptualization, writing – original draft. **Lary Souza Olegario**: conceptualization, writing – original draft, methodology. **Taliana Kênia Alencar Bezerra**: methodology, data curation, formal analysis. **Marta Suely Madruga**: methodology, data curation, formal analysis. **Fábio Anderson Pereira da Silva**: conceptualization, funding acquisition, writing – original draft, writing – review and editing, methodology, validation, project administration, data curation, supervision, resources.

## Conflicts of Interest

The authors declare no conflicts of interest.

## Supporting information




**Supplementary Material**: jfds71462‐sup‐0001‐SuppMat.docx

## Data Availability

Data will be made available on request.
