# Supplementary material for: Sensory Perceptions, Evoked Emotions, and Instrumental Parameters of Plant‐Based and Hybrid Meatballs Added With Fermented Cashew Apple Waste: A Multifactorial Approach
Source: J Food Sci. 2026 Sep 21;91(9):e71462. doi: 10.1111/1750-3841.71462 (PMC13594688; doi:10.1111/1750-3841.71462)
Supplement: Supplementary file 1 [file JFDS-91-0-s001.docx]

**Supplementary material**

Table S1: Formulations of analog and hybrid meatballs.

| **Component** | **Analog** | | | | **Hybrid** | | | |
| --- | --- | --- | --- | --- | --- | --- | --- | --- |
|  | **AC** | **A10** | **A20** | **A30** | **HC** | **H10** | **H20** | **H30** |
| Beef (chuck) | 0.0 | 0.0 | 0.0 | 0.0 | 60.9 | 54.8 | 48.7 | 42.6 |
| Water | 38.3 | 33.9 | 29.7 | 25.6 | 0.0 | 0.0 | 0.0 | 0.0 |
| Textured soybean protein | 21.7 | 19.3 | 16.9 | 14.5 | 0.0 | 0.0 | 0.0 | 0.0 |
| Enriched cashew apple flour | 0.0 | 5.9 | 11.6 | 17.2 | 0.0 | 6.1 | 12.2 | 18.3 |
| Soy sauce | 3.0 | 3.0 | 3.0 | 3.0 | 3.0 | 3.0 | 3.0 | 3.0 |
| Chia gel | 17.4 | 17.4 | 17.4 | 17.4 | 17.7 | 17.7 | 17.7 | 17.7 |
| Breadcrumbs | 11.1 | 11.1 | 11.1 | 11.1 | 11.2 | 11.2 | 11.2 | 11.2 |
| Soybean oil* | 5.2 | 6.5 | 7.9 | 9.2 | 3.7 | 3.7 | 3.7 | 3.7 |
| Smoked paprika | 0.7 | 0.7 | 0.7 | 0.7 | 0.7 | 0.7 | 0.7 | 0.7 |
| Onion powder | 0.7 | 0.7 | 0.7 | 0.7 | 0.7 | 0.7 | 0.7 | 0.7 |
| Garlic powder | 0.7 | 0.7 | 0.7 | 0.7 | 0.7 | 0.7 | 0.7 | 0.7 |
| Salt | 0.4 | 0.4 | 0.4 | 0.4 | 0.4 | 0.4 | 0.4 | 0.4 |
| Chimichurri sauce | 0.6 | 0.6 | 0.6 | 0.6 | 0.6 | 0.6 | 0.6 | 0.6 |
| Black pepper powder | 0.1 | 0.1 | 0.1 | 0.1 | 0.1 | 0.1 | 0.1 | 0.1 |
| Flavor enhancer | 0.1 | 0.1 | 0.1 | 0.1 | 0.1 | 0.1 | 0.1 | 0.1 |

HC: control hybrid meatball, H10: hybrid meatball prepared with 10% substitution of beef with enriched cashew apple flour, H20: hybrid meatball prepared with 20% substitution of beef with enriched cashew apple flour, H30: hybrid meatball prepared with 30% substitution of beef with enriched cashew apple flour. AC: control analog meatball, A10: analog meatball prepared with 10% substitution of soy protein with enriched cashew apple flour, A20: analog meatball prepared with 20% substitution of soy protein with enriched cashew apple flour, A30: analog meatball prepared with 30% substitution of soy protein with enriched cashew apple flour.

*In analog meatballs, for every 10% substitution of soy protein with enriched cashew apple flour, 10 g of soybean oil were added for technological purposes.

Table S2: Proximate composition and physicochemical parameters of analog and hybrid meatballs.

| **Parameters** | **Treatments** | | | | | | | |
| --- | --- | --- | --- | --- | --- | --- | --- | --- |
|  | **AC** | **A10** | **A20** | **A30** | **HC** | **H10** | **H20** | **H30** |
| Moisture | 62.67±0.80^A^ | 57.51±0.76^B^ | 55.72±0.70^B^ | 52.92±1.15^C^ | 62.48±0.19^w^ | 59.41±0.73^x^ | 55.69±1.1^y^ | 50.44±1.11^z^ |
| Protein | 11.15±0.50^A^ | 9.85±0.44^A^ | 10.81±1.47^A^ | 10.88±0.43^A^ | 16.83±0.50^w^ | 15.58± 1.02^w^ | 15.70±0.78^w^ | 16.24±2.05^w^ |
| Lipid | 6.31±0.70^A^ | 2.73±0.18^B^ | 2.38±0.71^B^ | 3.94±0.15^B^ | 3.53±1.16^y^ | 5.12±1.41^xy^ | 6.97±0.23^xw^ | 8.83±0.30^w^ |
| Ash | 2.14±0.07^A^ | 2.27±0.08^A^ | 2.27±0.10^A^ | 2.29±0.13^A^ | 2.01±0.09^x^ | 2.05±0.08^x^ | 2.34±0.11^w^ | 2.42±0.09^w^ |
| pH | 6.30±0.05^A^ | 6.01±0.03^B^ | 5.83±0.01^C^ | 5.69±0.02^D^ | 5.92±0.04^w^ | 5.63±0.06^x^ | 5.53±0.05^xy^ | 5.51±0.03^y^ |
| Total acidity | 0.48±0.04^C^ | 0.70±0.03^B^ | 0.86±0.04^A^ | 0.83±0.04^A^ | 0.37±0.07^x^ | 0.57±0.07^w^ | 0.60±0.05^w^ | 0.58±0.02^w^ |

Different capital letters (A-D) indicate significant differences of analog samples by Tukey test (P<0.05). Different lowercase letters (w-z) indicate significant differences of hybrid samples by Tukey test (P<0.05). HC: control hybrid meatball; H10: hybrid meatball prepared with 10% beef substitution using enriched cashew apple flour; H20: hybrid meatball prepared with 20% beef substitution using enriched cashew apple flour; H30: hybrid meatball prepared with 30% beef substitution using enriched cashew apple flour. AC: control analog meatball; A10: analog meatball prepared with 10% substitution of soy protein with enriched cashew apple flour; A20: analog meatball prepared with 20% substitution of soy protein with enriched cashew apple flour; A30: analog meatball prepared with 30% substitution of soy protein with enriched cashew apple flour.

Table S3: Instrumental color of analog and hybrid meatballs.

| **Treatments** | **Parameters** | | | | | |
| --- | --- | --- | --- | --- | --- | --- |
|  | **L*** | **a*** | **b*** | **ΔE** | **Visualization** |  |
| **AC** | 31.17±0.69^A^ | +4.00±0.48^A^ | +23.92±1.11^A^ | - |  |  |
| **A10** | 29.95±1.40^A^ | +2.18±0.28^B^ | +22.10±0.53^B^ | 2.85 |  |  |
| **A20** | 30.92±0.44^A^ | +4.27±0.47^A^ | +22.77±1.57^AB^ | 1.21 |  |  |
| **A30** | 31.23±0.65^A^ | +4.53±0.71^A^ | +24.37±0.71^A^ | 0.70 |  |  |
| **HC** | 26.08±0.52^x^ | +2.75±0.53^x^ | +23.18±1.07^w^ | - |  |  |
| **H10** | 28.75±0.41^y^ | +2.15± 0.24^x^ | +23.45±1.12^w^ | 2.82 |  |  |
| **H20** | 30.92±1.21^w^ | +2.55± 0.27^x^ | +23.77±1.09^w^ | 5.04 |  |  |
| **H30** | 30.85±0.92^w^ | +4.00± 0.45^w^ | +24.63±1.18^w^ | 5.53 |  |  |

Different capital letters (A-D) indicate significant differences of analog samples by Tukey test (P<0.05). Different lowercase letters (w-z) indicate significant differences of hybrid samples by Tukey test (P<0.05). HC: control hybrid meatball; H10: hybrid meatball prepared with 10% beef substitution using enriched cashew apple flour; H20: hybrid meatball prepared with 20% beef substitution using enriched cashew apple flour; H30: hybrid meatball prepared with 30% beef substitution using enriched cashew apple flour. AC: control analog meatball; A10: analog meatball prepared with 10% substitution of soy protein with enriched cashew apple flour; A20: analog meatball prepared with 20% substitution of soy protein with enriched cashew apple flour; A30: analog meatball prepared with 30% substitution of soy protein with enriched cashew apple flour.

Table S4: Texture Profile Analysis (TPA) of analog and hybrid meatballs.

| **Treatments** | **Parameters** | | | | |  |
| --- | --- | --- | --- | --- | --- | --- |
|  | **Hardness (N)** | **Springiness** | **Cohesiveness** | **Gumminess (N)** | **Chewiness (N)** | **Resilience** |
| **AC** | 13.03±1.41^A^ | 0.55±0.06^A^ | 0.37±0.02^A^ | 4.82±0.51^A^ | 2.68±0.48^A^ | 0.12±0.01^A^ |
| **A10** | 10.88±3.53^A^ | 0.45±0.06^B^ | 0.31±0.01^B^ | 3.41±1.20^A^ | 1.51±0.41^B^ | 0.10±0.00^B^ |
| **A20** | 17.36±5.16^A^ | 0.44±0.04^B^ | 0.31±0.01^B^ | 5.33±1.58^A^ | 2.32±0.55^AB^ | 0.10±0.01^B^ |
| **A30** | 14.48±4.95^A^ | 0.42±0.02^B^ | 0.26±0.04^C^ | 3.89±1.78^A^ | 1.61±0.69^B^ | 0.08±0.01^C^ |
| **HC** | 16.18±5.59^x^ | 0.58±0.06^w^ | 0.37±0.01^w^ | 6.03±2.08^x^ | 3.52±1.37^x^ | 0.13±0.00^w^ |
| **H10** | 20.64±7.75^x^ | 0.46±0.04^x^ | +0.33±0.03^wx^ | 6.81±2.33^x^ | 3.03±0.75^x^ | 0.11±0.02^xy^ |
| **H20** | 23.67±3.56^x^ | 0.40±0.04^y^ | 0.32±0.01^x^ | 7.45±1.06^x^ | 3.01±0.56^x^ | 0.10±0.00^y^ |
| **H30** | 43.56±10.95^w^ | 0.56±0.08^w^ | 0.37±0.03^w^ | 16.02±4.08^w^ | 8.81±1.98^w^ | 0.12±0.02^wx^ |

Different capital letters (A-D) indicate significant differences of analog samples by Tukey test (P<0.05). Different lowercase letters (w-z) indicate significant differences of hybrid samples by Tukey test (P<0.05). HC: control hybrid meatball; H10: hybrid meatball prepared with 10% beef substitution using enriched cashew apple flour; H20: hybrid meatball prepared with 20% beef substitution using enriched cashew apple flour; H30: hybrid meatball prepared with 30% beef substitution using enriched cashew apple flour. AC: control analog meatball; A10: analog meatball prepared with 10% substitution of soy protein with enriched cashew apple flour; A20: analog meatball prepared with 20% substitution of soy protein with enriched cashew apple flour; A30: analog meatball prepared with 30% substitution of soy protein with enriched cashew apple flour.

Table S5 **–** Sensory evaluation of analog and hybrid meatballs.

| **Parameter** | **Treatments** | | | | | |
| --- | --- | --- | --- | --- | --- | --- |
|  | **AC** | **A10** | **A20** | **HC** | **H10** | **H20** |
| **Appearance** | 6.90 ± 1.68^A^ | 6.95 ± 1.39^A^ | 6.90 ± 1.71^A^ | 6.90 ± 1.94^A^ | 6.45 ± 1.79^A^ | 6.20 ± 1.70^A^ |
| **Aroma** | 6.85 ± 1.46^AB^ | 6.20 ± 1.74^B^ | 6.00 ± 1.69^BC^ | 7.80 ± 1.24^A^ | 5.45 ± 1.96^BC^ | 4.50 ± 2.11^C^ |
| **Flavor** | 6.30 ± 1.45^B^ | 6.10 ± 1.92^B^ | 5.60 ± 1.85^B^ | 7.95 ± 1.50^A^ | 6.45 ± 1.90^AB^ | 5.20 ± 1.96^B^ |
| **Texture** | 6.80 ± 1.40^AB^ | 6.40 ± 2.01^AB^ | 6.30 ± 1.78^AB^ | 7.70 ± 1.17^A^ | 6.60 ± 1.64^AB^ | 5.80 ± 1.79^B^ |
| **Overall Impression** | 6.70 ± 1.42^AB^ | 6.30 ± 1.66^BC^ | 5.95 ± 1.67^BC^ | 7.95 ± 1.23^A^ | 6.25 ± 1.65^BC^ | 5.25 ± 1.62^C^ |

Different capital letters in the same line indicate significant differences among samples by Tukey test (P<0.05). HC: control hybrid meatball; H10: hybrid meatball prepared with 10% beef substitution using enriched cashew apple flour; H20: hybrid meatball prepared with 20% beef substitution using enriched cashew apple flour. AC: control analog meatball; A10: analog meatball prepared with 10% substitution of soy protein with enriched cashew apple flour; A20: analog meatball prepared with 20% substitution of soy protein with enriched cashew apple flour.

Table S6: **-** Descriptors clarified by consumers during PAE session.

| **Groups** | **Attributes** |
| --- | --- |
| Appearance | Brown color^1,2,3^, Uniformity^1,2,3,4^, Luminosity^2,3,4,5,6^, Dry appearance^3,4,5,6^ |
| Aroma | Meat^1^, Seasoned^1,2,3^, Mealy^4,5,6^, Cashew^4,5,6^, Fermented^6^ |
| Flavor | Seasoned^1,2^, Spicy^1,2,3,4,5,6^, Soy^4,5,6^, Rancidity^5,6^, Fermented cashew^6^ |
| Texture | Juiciness^1^, Firmness^1,2,3,4,5^, Crispness^1,2,3,4,5,6^, Fibrous^3,4,5,6^, Gumminess^4,5,6^ |
| Total | 19 |

Order of importance for product acceptance. 1=most important, 5 = least important. Identical numbers indicate attributes considered equally important by consumers. Differences observed using Friedman test (P < 0.05).

Tabel S7. Sensory responses to evoked emotions in analog and hybrid meatballs.

| **Attribute** | **AC** | **A-EF** | **HC** | **H-EF** | **p-value** |
| --- | --- | --- | --- | --- | --- |
|  | 3 | 0 | 1 | 0 | 0.112 |
|  | 3 | 4 | 4 | 0 | 0.217 |
|  | 8^a^ | 2^b^ | 8^a^ | 1^b^ | **0.021** |
|  | 3 | 0 | 1 | 0 | 0.112 |
|  | 22^b^ | 27^b^ | 24^b^ | 62^a^ | **<0.0001** |
|  | 3 | 3 | 0 | 3 | 0.356 |
|  | 7 | 10 | 8 | 13 | 0.403 |
|  | 4 | 5 | 5 | 8 | 0.587 |
|  | 14^b^ | 27^a^ | 14^b^ | 24^a^ | **0.027** |
|  | 12 | 9 | 4 | 6 | 0.118 |
|  | 17 | 11 | 11 | 5 | 0.062 |
|  | 0 | 2 | 1 | 0 | 0.300 |
|  | 16^ab^ | 5^b^ | 20^a^ | 4^b^ | **0.000** |
|  | 8 | 5 | 6 | 6 | 0.809 |
|  | 4 | 3 | 5 | 4 | 0.896 |
|  | 1 | 0 | 1 | 0 | 0.572 |
|  | 3 | 1 | 5 | 2 | 0.305 |
|  | 3 | 1 | 1 | 0 | 0.284 |
|  | 5 | 8 | 13 | 12 | 0.119 |
|  | 16^a^ | 8^ab^ | 14^a^ | 1^b^ | **0.001** |
|  | 15^a^ | 11^a^ | 15^a^ | 3^b^ | **0.022** |
|  | 17 | 27 | 15 | 16 | 0.089 |
|  | 0 | 0 | 1 | 0 | 0.392 |
|  | 2 | 1 | 0 | 0 | 0.300 |
|  | 5 | 2 | 5 | 1 | 0.224 |
|  | 11 | 10 | 7 | 11 | 0.715 |
|  | 25^ab^ | 32^a^ | 25^ab^ | 11^b^ | **0.003** |
|  | 7 | 3 | 4 | 8 | 0.319 |
|  | 27 | 33 | 23 | 31 | 0.372 |
|  | 2 | 0 | 1 | 0 | 0.300 |
|  | 4 | 1 | 1 | 1 | 0.234 |
|  | 5 | 2 | 4 | 0 | 0.147 |
|  | 12^a^ | 17^a^ | 5^b^ | 14^a^ | **0.047** |

AC: control analog meatball; A-EF: analog meatball added with enriched cashew apple flour; HC: control hybrid meatball; H-EF: hybrid meatball added with enriched cashew apple flour. Citation frequencies followed by different lowercase letters in the same line indicate differences between packaging using the Cochran Test and McNemar (Bonferroni) test (P < 0.05).

Table S8. Demographic characteristics of participants for each sensory technique employed.

| **Sensory approach** | **Evaluated product** | **Participants (n)** | **Female (%)** | **Male (%)** | **Transgender (%)** | **18–25 years (%)** | **26–35 years (%)** | **36–45 years (%)** | **46–55 years (%)** | **>55 years (%)** |
| --- | --- | --- | --- | --- | --- | --- | --- | --- | --- | --- |
| FCP | Plant-based meatballs | 26 | 64 | 36 | 0 | 20 | 56 | 24 | 0 | 0 |
| FCP | Hybrid meatballs | 24 | 48 | 52 | 0 | 19 | 57 | 24 | 0 | 0 |
| PAE | Plant-based meatballs and Hybrid meatballs | 20 | 55 | 40 | 5 | 25 | 60 | 10 | 0 | 5 |
| Emotional response evaluation | Plant-based | 100 | 44 | 53 | 3 | 22 | 63 | 12 | 1 | 2 |
| Emotional response evaluation | Hybrid | 100 | 61 | 37 | 2 | 33 | 48 | 11 | 5 | 3 |

Figure S1. Preference Order test for analog (A) and hybrid (B) meatballs.

**(A)	(B)**

Figure S2. Purchase intention of analog (A) and hybrid (B) meatballs.

**(A)	(B)**

Figure S3 - Relevant emojis used in validated methodology for evoked emotion related to food. (Cruz *et al.,* 2021).
